# One-year mortality of colorectal cancer patients: development and validation of a prediction model using linked national electronic data

**DOI:** 10.1038/s41416-020-01034-w

**Published:** 2020-08-24

**Authors:** Thomas E. Cowling, Alexis Bellot, Jemma Boyle, Kate Walker, Angela Kuryba, Sarah Galbraith, Ajay Aggarwal, Michael Braun, Linda D. Sharples, Jan van der Meulen

**Affiliations:** 1 Clinical Effectiveness Unit, Royal College of Surgeons of England, London, UK; 2 Department of Health Services Research and Policy, London School of Hygiene & Tropical Medicine, London, UK; 3 Department of Applied Mathematics and Theoretical Physics, University of Cambridge, Cambridge, UK; 4 Alan Turing Institute, London, UK; 5 Department of Palliative Care, Addenbrooke’s Hospital, Cambridge University Hospitals NHS Foundation Trust, Cambridge, UK; 6 Department of Clinical Oncology, Guy’s Hospital, Guy’s and St Thomas’ NHS Foundation Trust, London, UK; 7 Department of Medical Oncology, The Christie NHS Foundation Trust, Manchester, UK; 8 Department of Medical Statistics, London School of Hygiene & Tropical Medicine, London, UK

**Keywords:** Outcomes research, Colorectal cancer

## Abstract

**Background:**

The existing literature does not provide a prediction model for mortality of all colorectal cancer patients using contemporary national hospital data. We developed and validated such a model to predict colorectal cancer death within 90, 180 and 365 days after diagnosis.

**Methods:**

Cohort study using linked national cancer and death records. The development population included 27,480 patients diagnosed in England in 2015. The test populations were diagnosed in England in 2016 (*n* = 26,411) and Wales in 2015–2016 (*n* = 3814). Predictors were age, gender, socioeconomic status, referral source, performance status, tumour site, TNM stage and treatment intent. Cox regression models were assessed using Brier scores, *c*-indices and calibration plots.

**Results:**

In the development population, 7.4, 11.7 and 17.9% of patients died from colorectal cancer within 90, 180 and 365 days after diagnosis. T4 versus T1 tumour stage had the largest adjusted association with the outcome (HR 4.67; 95% CI: 3.59–6.09). *C*-indices were 0.873–0.890 (England) and 0.856–0.873 (Wales) in the test populations, indicating excellent separation of predicted risks by outcome status. Models were generally well calibrated.

**Conclusions:**

The model was valid for predicting short-term colorectal cancer mortality. It can provide personalised information to support clinical practice and research.

## Background

In 2018, colorectal cancer was the third most incident cancer and caused the second largest number of cancer deaths in high-income countries.^1,2^ It is a heterogeneous disease with varied presentations and large differences in prognosis. Considering the cancer stage alone, 1-year net survival for localised and metastatic cancer varies from 96 to 55%, respectively, in the United States.^3^

Clinical prediction models combine multiple prognostic factors to estimate individualised risks of outcomes for each patient.^4,5^ These risk predictions have many uses. In colorectal cancer research, prediction models have been used to examine prognosis in clinical trials,^6^ to control for confounding in observational studies,^7^ and to assess the added prognostic value of biomarkers.^8^ In clinical practice, they may be used to inform treatment decisions and to communicate prognosis to patients, in line with the aims of personalised medicine and shared decision-making.^9^

In the absence of high-quality prediction models, clinicians’ predictions of cancer survival may be inaccurate, non-transparent, and difficult to explain to patients.^10–12^ Existing models for colorectal cancer mortality have focused on selected populations recruited to clinical trials (such as stage III and IV groups),^6,13,14^ risks after surgery or chemotherapy,^15,16^ or long-term survival using primary care data.^17^ A recent systematic review^18^ did not identify any models to predict mortality for all colorectal cancer patients using contemporary national hospital data.

In this study, our objective was to develop and validate a prediction model for death from colorectal cancer within 3, 6 and 12 months after diagnosis. To do this, we analysed national electronic hospital records linked to official mortality data from England and Wales.

## Methods

### Study populations

The National Bowel Cancer Audit collects data on adults (aged 18 years or over) newly diagnosed with colorectal cancer (International Classification of Diseases 10th Revision (ICD-10) codes: C18-20^19^) in England and Wales.^20^ These data are entered into electronic record systems by hospital staff and later combined into a pooled national dataset by the National Health Service (NHS). We analysed data for patients whose date of diagnosis was from January 2015 to December 2016.

We defined one population to develop the prediction model and two separate populations to test the performance of this model. The eligible population used for model development included patients who were diagnosed in England in 2015 (*n* = 28,505 patients). The first test population included patients who were diagnosed in England in 2016 *(n* = 28,216 patients). The second test population included patients who were diagnosed in Wales in 2015 or 2016 (*n* = 3861 patients).

### Outcome

The outcome was death from colorectal cancer as identified from official death records provided by the Office for National Statistics.^21^ We defined death from colorectal cancer using relevant ICD-10 codes recorded as the ‘underlying cause of death’ (see Supplement S1). The underlying cause is ‘the disease or injury, which initiated the train of morbid events leading directly to death’.^22^

Time to death was defined as the number of days between the date of diagnosis (as recorded in the National Bowel Cancer Audit dataset) and the date of death from colorectal cancer (as recorded in Office for National Statistics mortality data). The date of diagnosis was ‘the date when cancer was confirmed or diagnosis agreed’, which is typically the date of the pathology report that confirmed cancer. Patients who died from other causes were censored on the date of death. Patients alive as of 1 January 2018 were censored on that date, providing at least 365 days of follow-up for all patients.

Records from the National Bowel Cancer Audit and Office for National Statistics datasets were combined using deterministic linkage based on each patient’s unique NHS number, date of birth, gender and postcode. From the 60,582 eligible patients (in both development and test sets), the final sample size was 57,705 patients (27,480 in the development population; 26,411 in the England test population; and 3814 in the Wales test population). Supplement S2 provides the sample flow chart. Distributions of variables were similar for the linked and unlinked patients (Supplement S3).

### Predictor variables

We used ten variables from the National Bowel Cancer Audit dataset as predictor variables: age, gender, socioeconomic status, source of referral, performance status, tumour site, TNM (tumour, node, metastasis) stage at diagnosis and treatment intent. All variables were recorded in electronic data systems around the time of the first meeting between clinicians to discuss patients’ treatment after diagnosis. We selected these predictors a priori to include variables recorded around the time of diagnosis that had relatively complete data (≥80% of values nonmissing).

Patient age was coded as a continuous variable defined as the number of complete years between the dates of birth and diagnosis. Gender was male/female. Socioeconomic status was defined as the national rank of a patient’s area of residence according to the Index of Multiple Deprivation;^23^ the mean population size of these areas was 1500.^23^ To aid interpretation, these ranks were linearly rescaled to have a median of zero and lower and upper quartiles of −1 and +1, respectively.^24^

The source of referral for investigation of suspected cancer had five categories: emergency hospital admission, urgent care/emergency department visit, primary care, national screening programme and ‘other’ (e.g. a separate outpatient clinic). Performance status was defined by five categories of the Eastern Cooperative Oncology Group score (ranging from ‘fully active’ to ‘completely disabled’).^25^ Tumour site was one of nine ICD-10 codes indexed under C18-20. T, N and M stages of the cancer were defined by the TNM Classification of Malignant Tumours 5th Edition.^26^ The treatment intent had three categories: curative, non-curative and no active cancer treatment.

All ten predictor variables were defined using the National Bowel Cancer Audit dataset. The original (incomplete) data were used to calculate descriptive statistics for each variable. To account for missing values of predictors, we used multiple imputation with chained equations to generate 40 complete datasets (see Supplement S4 for details). All analysis of associations between the outcome and predictors was done using these 40 imputed datasets. We pooled model estimates and performance measures across the datasets to produce the final results.^27^

### Statistical analysis

We used Cox proportional hazards regression^28^ to estimate associations between predictor variables and the hazard of colorectal cancer death. Deaths from other causes were treated as censoring events. All predictors entered the regression model simultaneously. We fitted linear associations with the outcome for age and socioeconomic status, as nonlinear transformations fitted by a multivariable fractional polynomial algorithm^29–31^ were well approximated by linear relationships.

We assessed model performance at 90, 180 and 365 days after diagnosis. Overall model performance was measured using Brier scores.^32^ These scores were calculated from the mean squared differences between predicted probabilities of colorectal cancer death within a given time period and the observed death status. We scaled these scores from 0–100% (0% if non-informative and 100% if perfect).^33^

To assess discrimination, we calculated the *c*-index.^34^ This indicates the proportion of all pairs of patients whose survival times could be ordered such that the patient with the lower predicted risk of colorectal cancer death survived longer.^24^ *C*-indices equal one for perfect models and 0.5 for random predictions. To assess model calibration, we plotted the predicted risks of colorectal cancer death against the actual observed risks, using the loess smoother to estimate the calibration curve.^24^

We assessed the internal validity of the model using 10-fold cross-validation and calculated mean values of the performance measures across the ten folds. We tested the performance of the model in two other populations: patients diagnosed in England in 2016 and in Wales in either 2015 or 2016.

### Sensitivity analyses

Three sensitivity analyses tested the specification of the model and its performance, as detailed in Supplement S5. These added interaction terms between key predictors, added a comorbidity score and the number of unplanned admissions in the past year as predictor variables, and assessed whether censoring of surviving patients at 365 days affected the associations estimated.

Data preparation was done using Stata (v15) and R (v3.5) was used for all statistical analysis.

## Results

In the population used to develop the prediction model, the percentages of patients who died from colorectal cancer were 7.4% (within 90 days), 11.7% (180 days) and 17.9% (365 days). These percentages were similar in the England test population but slightly greater in the Wales test population (Table 1). The Wales population had greater percentages of patients who were referred for diagnostic investigations after an emergency admission (29.0% vs. 13.0% in the development population) and who had metastases (25.6% vs. 22.1%). Most patients in each population were treated with curative intent (73.3–74.1%) (Table 2).

**Table 1:** Descriptive statistics for the outcome variable and follow-up time.

	Development population	Test population—England	Test population—Wales
Number of patients	27,480	26,411	3814
CRC death within, *n* (%)			
90 days	2024 (7.4)	1862 (7.1)	362 (9.5)
180 days	3210 (11.7)	2978 (11.3)	540 (14.2)
365 days	4926 (17.9)	4659 (17.6)	820 (21.5)
Other death within, *n* (%)			
90 days	483 (1.8)	467 (1.8)	83 (2.2)
180 days	762 (2.8)	677 (2.6)	117 (3.1)
365 days	1139 (4.1)	1039 (3.9)	158 (4.1)
Median follow-up (IQR), days^a^	908 (818–999)	544 (452–636)	705 (527–906)

*CRC* colorectal cancer, *IQR* interquartile range.

^a^Calculated using the reverse Kaplan–Meier method.

**Table 2:** Descriptive statistics for predictor variables.

	Development population (*n* = 27,480)	Test population—England (*n* = 26,411)	Test population—Wales (*n* = 3814)
Median age (years; IQR)^a^	72 (63–80)	72 (63–80)	72 (64–80)
Gender, *n* (%)			
Male (*vs*. female)	15,505 (56.4)	15,101 (57.2)	2209 (57.9)
Missing	7	12	0
Median socioeconomic status (IQR)^b^	0.2 (−0.8 to 1.1)	0.3 (−0.7 to 1.1)	-1.9 (−1.9 to -1.8)^c^
Referral source, *n* (%)			
Emergency admission	3542 (13.0)	3224 (12.4)	1092 (29.0)
Urgent care/ED visit	741 (2.7)	768 (3.0)	55 (1.5)
Screening	2701 (10.0)	2749 (10.6)	398 (10.6)
Primary care	15,227 (56.1)	14,549 (56.0)	1871 (49.6)
Other	4943 (18.2)	4689 (18.1)	354 (9.4)
Missing	326	432	44
Performance status, *n* (%)^d^			
0 (fully active)	10,014 (43.9)	10,234 (45.8)	1217 (42.5)
1	7166 (31.4)	6770 (30.3)	924 (32.2)
2	3505 (15.4)	3285 (14.7)	462 (16.1)
3	1793 (7.9)	1729 (7.7)	234 (8.2)
4 (completely disabled)	340 (1.5)	313 (1.4)	29 (1.0)
Missing	4662	4080	948
Tumour site, *n* (%)			
Caecum	4087 (14.9)	3885 (14.7)	574 (15.1)
Ascending colon	3043 (11.1)	2908 (11.0)	416 (10.9)
Hepatic flexure	1083 (3.9)	1059 (4.0)	167 (4.4)
Transverse colon	1783 (6.5)	1725 (6.5)	232 (6.1)
Splenic flexure	688 (2.5)	671 (2.5)	104 (2.7)
Descending colon	946 (3.4)	980 (3.7)	118 (3.1)
Sigmoid colon	6364 (23.2)	6189 (23.4)	842 (22.1)
Rectosigmoid junction	1545 (5.6)	1477 (5.6)	226 (5.9)
Rectum	7941 (28.9)	7517 (28.5)	1135 (29.8)
Missing	0	0	0
T-stage, *n* (%)			
1	1338 (6.1)	1291 (6.0)	142 (4.3)
2	4387 (20.0)	4275 (20.0)	630 (19.2)
3	11,399 (51.9)	11,081 (51.8)	1670 (50.9)
4	4847 (22.1)	4731 (22.1)	840 (25.6)
Missing	5509	5033	532
N-stage, *n* (%)			
0	10,790 (47.8)	10,554 (48.0)	1532 (46.1)
1	7657 (33.9)	7566 (34.4)	1108 (33.4)
2/3	4137 (18.3)	3865 (17.6)	682 (20.5)
Missing	4896	4426	492
M-stage, *n* (%)			
0	17,975 (77.9)	17,853 (78.6)	2413 (74.4)
1	5094 (22.1)	4861 (21.4)	832 (25.6)
Missing	4411	3697	569
Treatment intent, *n* (%)			
Curative	19,078 (73.3)	18,089 (73.7)	2406 (74.1)
Non-curative	5440 (20.9)	4869 (19.8)	722 (22.2)
No active cancer treatment	1508 (5.8)	1601 (6.5)	118 (3.6)
Missing	1454	1852	568

Number of patients with no missing values across all populations = 35,472 (61.5%).

*ED* emergency department, *IQR* interquartile range.

^a^Age range was 18–104.

^b^Rescaled national rank of the area in which a patient resided (0 is the median; −1 is the lower quartile, more deprived; 1 is the upper quartile, less deprived).

^c^Values not comparable to England values due to differences in scales

^d^Measured on the Eastern Cooperative Oncology Group (ECOG) scale.

Missing values were most common for the performance status of the patient (16.8%) and the T and N-stages of the cancer (19.2% and 17.0%; Table 2). Data fields were complete across all variables for 61.5% of patients. These patients were more likely to be treated with curative intent (76.6% vs. 67.5%) and to survive until the end of follow-up (70.6% vs. 61.5%) than patients who had at least one predictor variable with a missing value (Supplement S6).

After multiple imputation of missing values, risks of colorectal cancer death were greatest for patients who had metastatic disease, had a treatment plan with non-curative intent or no active cancer treatment, or had an unfavourable performance status (Table 3). The risk of cancer death within 365 days was more than 50% for three patient groups: patients in the two worst performance status categories (50.3% and 58.3%) and patients with a non-curative treatment intent (51.9%).

**Table 3:** Univariable and multivariable associations between the outcome and predictor variables in the development population, estimated using Cox regression.

	CRC death within 90 days (%)	CRC death within 180 days (%)	CRC death within 365 days (%)	Univariable associations, HR (95% CI)	Multivariable associations, HR (95% CI)
Age (per 10 years)	–	–	–	1.39 (1.37–1.42)	1.21 (1.18–1.23)
Gender					
Male	6.8	11.0	16.8	1	1
Female	8.0	12.6	19.3	1.09 (1.05–1.14)	1.02 (0.98–1.07)
Socioeconomic status^a^	–	–	–	0.92 (0.90–0.93)	0.96 (0.95– 0.98)
Referral source					
Emergency admission	18.1	25.0	34.5	1	1
Primary care	6.0	10.3	16.8	0.47 (0.44–0.49)	0.73 (0.69–0.78)
Urgent care/ED visit	15.6	22.0	31.1	0.88 (0.78–0.99)	0.98 (0.86–1.12)
Screening	0.7	1.3	2.2	0.09 (0.08–0.11)	0.33 (0.29–0.39)
Other	6.2	10.4	16.3	0.46 (0.43–0.49)	0.75 (0.69–0.81)
Performance status^b^					
0 (fully active)	2.3	4.5	8.5	1	1
1	5.2	9.3	15.9	1.73 (1.63–1.84)	1.20 (1.13–1.28)
2	11.3	17.8	26.9	2.95 (2.76–3.15)	1.53 (1.42–1.66)
3	28.3	39.7	50.3	6.99 (6.51–7.51)	2.34 (2.14–2.55)
4 (completely disabled)	39.7	48.8	58.3	10.04 (8.68–11.60)	3.37 (2.87–3.96)
Tumour site					
Caecum	11.0	16.7	25.1	1	1
Ascending colon	7.8	12.4	19.9	0.77 (0.71–0.84)	0.92 (0.84–1.00)
Hepatic flexure	9.9	15.4	23.4	0.92 (0.82–1.03)	1.14 (1.01–1.29)
Transverse colon	10.0	16.3	23.0	0.89 (0.81–0.98)	1.08 (0.98–1.19)
Splenic flexure	9.3	12.5	18.2	0.73 (0.63–0.84)	0.88 (0.76–1.03)
Descending colon	10.3	14.2	18.9	0.77 (0.68–0.88)	0.92 (0.80–1.05)
Sigmoid colon	6.7	10.7	16.1	0.66 (0.62–0.71)	0.81 (0.75–0.87)
Rectosigmoid junction	7.3	11.9	18.3	0.73 (0.66–0.81)	0.85 (0.76–0.94)
Rectum	4.5	7.7	12.9	0.61 (0.57–0.65)	0.79 (0.73–0.85)
T-stage					
1	0.8	1.4	2.1	1	1
2	2.5	3.8	6.1	2.93 (2.24–3.84)	2.03 (1.55–2.66)
3	6.2	9.9	15.8	7.30 (5.66–9.41)	3.02 (2.32–3.93)
4	16.0	25.2	37.2	17.80 (13.80–22.96)	4.67 (3.59–6.09)
N-stage					
0	4.2	6.5	10.4	1	1
1	8.4	13.4	20.5	1.98 (1.87–2.10)	1.15 (1.08–1.23)
2/3	13.5	21.5	32.1	3.34 (3.15–3.56)	1.52 (1.42–1.64)
M-stage					
0	3.0	5.0	8.7	1	1
1	22.6	34.9	49.9	7.45 (7.09–7.82)	2.81 (2.62–3.02)
Treatment intent					
Curative	1.5	2.7	5.9	1	1
Non-curative	23.0	36.3	51.9	10.74 (10.22–11.28)	3.85 (3.60–4.11)
No active cancer treatment	23.8	34.1	45.3	8.41 (7.80–9.06)	3.85 (3.52–4.21)

*CI* confidence interval, *CRC* colorectal cancer, *ED* emergency department, *HR* hazard ratio.

^a^National rank of the area in which a patient resided (1 unit increase equals difference between quartiles of ranks).

^b^Measured on the Eastern Cooperative Oncology Group (ECOG) scale.

In the multivariable model including all predictor variables, the greatest relative difference in the hazard of colorectal cancer death was between the T4 and T1 stages (hazard ratio (HR) = 4.67; 95% confidence interval (CI): 3.59–6.09). Compared to patients with a curative treatment intent, the hazard of colorectal cancer death was 3.85 times greater for patients whose treatment plan was non-curative (HR = 3.85, 95% CI: 3.60–4.11) or did not include active cancer treatment (HR = 3.85, 95% CI: 3.52–4.21). Outcomes were similar between the non-curative and no active cancer treatment groups (HR = 1.00, 95% CI: 0.92–1.09) (Table 3).

Predicted probabilities of colorectal cancer death varied widely within treatment intent categories. In the England test population, the 10th and 90th percentiles of predicted risks within 365 days were 1.7% and 12.9% for patients treated with curative intent, 23.8% and 88.8% for patients with a non-curative intent and 16.3% and 89.6% for patients with no active cancer treatment planned.

### Model performance

**Fig. 1:**
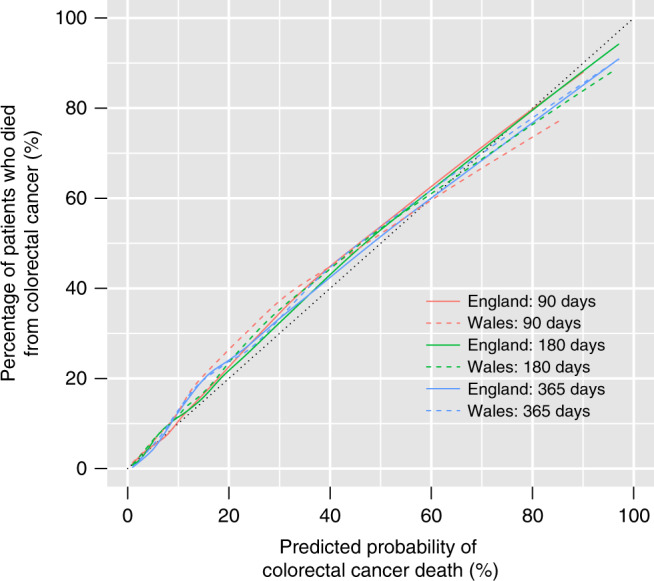
Calibration plots for predicted probabilities of colorectal cancer death within 90, 180 and 365 days after diagnosis, in the England and Wales test populations. Note: The coloured lines represent the smoothed relationships between the observed and predicted risks of colorectal cancer death. The black dotted 45° line represents the ideal relationship showing perfect calibration.

The probabilities of colorectal cancer death predicted by the model were well calibrated with the observed proportions of patients that died, in both England and Wales test populations (Fig. 1).

**Fig. 2:**
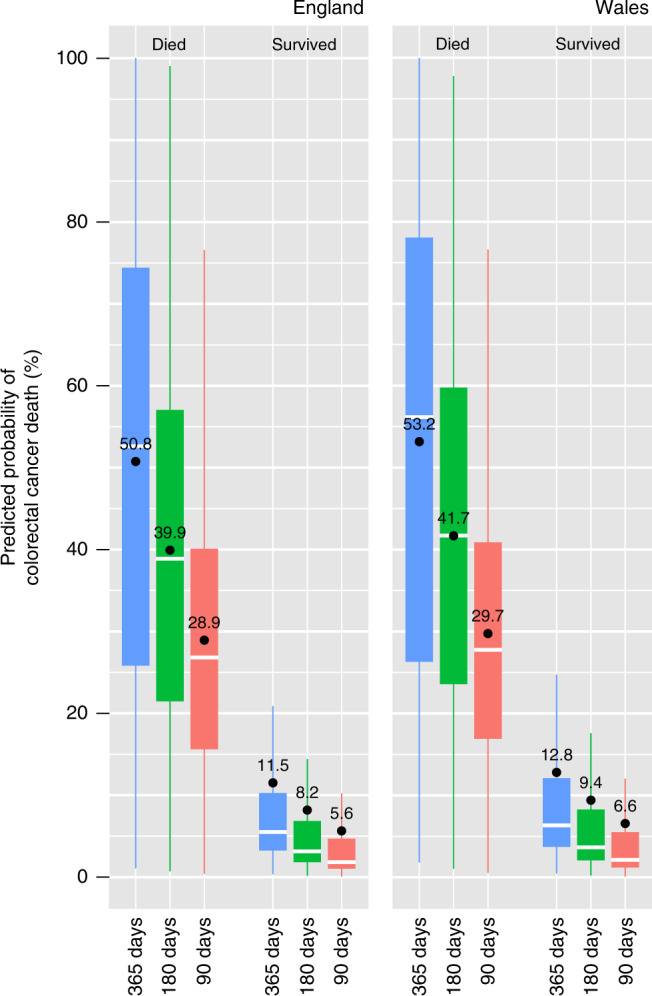
Boxplots comparing predicted probabilities of colorectal cancer death by outcome status within 90, 180 and 365 days after diagnosis, in the England and Wales test populations. Note: Boxes are drawn from the lower to upper quartile of predicted probabilities with a white horizontal line at the median value. Annotated values and black dots correspond to mean values. Whiskers are drawn to the most extreme predicted probabilities that are no more than 1.5 times the interquartile range from the box.

The model typically predicted very low risks of colorectal cancer death for patients who did not experience this outcome (Fig. 2). The predicted risks were generally much greater for patients who did die from colorectal cancer, particularly for the 365-day outcome period. As a result, the predicted probabilities of colorectal cancer death were well separated between patients who did and did not have this outcome (Fig. 2). This was reflected in large values of the *c*-index, ranging from 0.873 to 0.890 and 0.856 to 0.873 in the England and Wales test populations, respectively (Table 4).

**Table 4:** Overall model performance and discrimination in the development and test populations.

	Development population	Test population—England	Test population—Wales
Scaled Brier score (%)			
90 days	23.5	23.7	24.1
180 days	32.6	32.4	32.1
365 days	39.5	40.0	40.1
*c*-index			
90 days	0.885	0.890	0.873
180 days	0.882	0.885	0.867
365 days	0.870	0.873	0.856

Scaled Brier scores range from 0 to 100% with larger scores indicating better performance.

*C*-indices range from 0.5 (random predictions) to 1 (perfect discrimination).

The overall performance of the model as measured by the scaled Brier score was best for the 365-day period, followed by the 180 then 90-day periods (Table 4). For the 365-day period in the England test population, the Brier score was improved by 40.0% compared to if the overall risk of colorectal cancer death had been used as the predicted probability for all patients, indicating a large improvement in prediction ability when using the model (versus no model).

### Sensitivity analyses

In the sensitivity analyses, interaction terms between patient age, M-stage and treatment intent did not improve model performance (maximum absolute difference in *c*-index or Brier score vs. main analysis = 0.001). Results were also similar when each patient’s history of comorbidities and unplanned hospitalisations were added as predictors (maximum absolute difference = 0.002). When patients who were alive 365 days after diagnosis were censored at this timepoint, predictor effects were similar to those in the main analysis (range of relative differences in HRs: 0.97–1.08).

## Discussion

The model developed was valid for predicting death from colorectal cancer within 3, 6, and 12 months after diagnosis in England and Wales. The model discriminated very well between patients who did and did not die from colorectal cancer, such that the former group typically had much higher predicted probabilities of death. These predictions were well calibrated with observed outcomes. The T-stage of the tumour had the largest adjusted association with the risk of death, followed by the treatment intent and performance status of the patient.

No single variable alone had a high positive predictive value for colorectal cancer death. For example, just over half of patients (51%) who did not have a curative treatment intent died within 365 days. Predicted risks of death varied widely across patients who did not have a curative intent. This wide variation also existed for patients who did have a curative treatment plan.

### Strengths and limitations

We used large, national datasets to develop a new model and examine its temporal and geographic validity in whole populations from two countries. The data used for predictor variables were entered as part of routine care processes and therefore represent information available to clinicians in practice around the time of decision-making. We used cause of death information from official death records to distinguish colorectal cancer deaths from other deaths, and we were able to measure these outcomes for at least 1 year after diagnosis for all patients. Although the patients in the test sets were similar to those in the development set, the differences in the type of referrals and TNM stages between England and Wales provided a reasonable test of external validity.

The model would likely be improved if further information about the cancer was available, such as the sites of any metastases or possibly molecular data, as well as additional characteristics of patients (such as frailty) and their cancer care. This may help to predict greater probabilities of colorectal cancer death for patients who experienced this outcome. Some uncertainty in prognosis may reflect the biological development of cancer and the possibility of treatment-related complications.

Detailed assessment of patients’ overall morbidity, particularly for older patients, could be used to contextualise predictions of cancer mortality in terms of overall life expectancy. However, the overall risk of dying from causes other than colorectal cancer within 1 year after diagnosis was only 4%, so other causes of mortality in this period may be less relevant to treatment decisions for most patients.

Differences in data collection or population characteristics may limit the generalisability of the model to other countries. Estimates of 1-year survival for colorectal cancer can differ markedly between high-income countries, such as 78% in England and 84% in Sweden in 2010–2012.^35^ The model may need to be recalibrated when used elsewhere if the survival differences are unexplained by differences in the distributions of predictors. However, despite survival in Wales being somewhat worse than in England in the current study, model calibration remained acceptable. Most predictors used have standard international definitions. We rescaled the measure of socioeconomic status so that it might approximate similarly rescaled measures in other settings.

In order to avoid the possibility that any racial biases in access to treatment are reinforced by the prediction model, we did not consider patient ethnicity as a predictor.^36^ This is in line with most clinical prediction models.^37^ Prognostic factors such as lymphovascular invasion, surgical margin status and definitive treatment were not included in the model as they are typically unknown around the time of diagnosis and were not relevant to all patients (some of whom do not receive surgery).

Missing data will have biased results if data were ‘missing not at random’, which multiple imputation cannot address. The extent of this bias cannot be ascertained from observed data, but each predictor had less than 20% of values missing, thus reducing the potential bias. National Bowel Cancer Audit records could not be linked to Office for National Statistics death records for 4.4% of eligible patients; distributions of predictor variables were similar between the linked and unlinked groups of patients but some bias due to linkage problems cannot be ruled out.

The 5th edition of the TNM system used in the analysis has been superseded by the 8th edition in the U.K., which will affect the N-stage of some (but relatively few) patients.

### Relation to existing literature

A previous study^17^ used primary care records and cancer registry data to develop a prediction model for longer-term survival (1, 5 and 10-year) of colorectal cancer patients in England. This model did not include several variables that are routinely recorded in clinical team meetings shortly after diagnosis such as the referral source, performance status, separate TNM stages and treatment intent. The *c*-index of 0.873 attained by our model for predicting 365-day cancer mortality in England is much greater than that reported for one-year mortality (from all causes) in the previous study (0.795 for men and 0.807 for women^17^). This indicates a large increase in performance (closer to the perfect *c*-index of 1), especially as *c*-indices are relatively insensitive to improvements in model fit.^38^

A systematic review^18^ reported several prediction models developed for mortality in subgroups of colorectal cancer patients, such as patients with stage III^6^ or metastatic^13,14^ cancer, or for posttreatment mortality.^15,16^ None of these models were developed to predict mortality for all colorectal cancer patients using contemporary national hospital data. A previous study^7^ by our group used linked National Bowel Cancer Audit and Office for National Statistics death records to develop a risk-adjustment model for 90-day postoperative mortality. This model used similar predictors to the model presented here and showed good discrimination (*c*-index = 0.799) and calibration; the *c*-index may have been lower in this surgical cohort partly due to the population being more homogeneous.

### Implications for research and practice

The predictor information used in the model is recorded electronically as part of routine practice in England and Wales, typically during clinical team meetings where patient care is planned. Patients’ risks of death within 3, 6 and 12 months could be automatically calculated in these meetings without additional data entry. Supplement S7 gives the formula for calculating predicted probabilities of colorectal cancer death within 90, 180 and 365 days after diagnosis.

The external validity of the model should be tested further before being used outside of England and Wales, possibly in combination with well-established methods for updating prediction models when used in new settings.^39^ Ideally, the effects of the model on decision-making and patient outcomes would also be evaluated in future research (though such impact studies are rare^40^).

The model’s predictions could be used to provide accurate prognostic information to patients, so that they can make informed decisions together with clinicians. The risk predictions may also help to prioritise patients for specialist palliative care services,^41,42^ given the wide range of predicted risks for patients without a curative treatment intent. The predictions also varied widely for those with a curative intent, which may help to inform the intensity of related treatment. Finally, the model could also be relevant to various clinical, epidemiological and biomarker studies.

## Supplementary information

Supplementary Information

## Data Availability

The data used in this study are available from NHS Digital and Public Health England’s Office for Data Release but restrictions apply to the availability of these data, which were used under license for the current study, and so are not publicly available. We do not have permission to share the patient-level records used in our analysis.
