# Supplementary material for: One-year mortality of colorectal cancer patients: development and validation of a prediction model using linked national electronic data
Source: Br J Cancer. 2020 Aug 24;123(10):1474–80. doi: 10.1038/s41416-020-01034-w (PMC7652941; doi:10.1038/s41416-020-01034-w)
Supplement: Supplementary file 1 — Supplementary Information [file 41416_2020_1034_MOESM1_ESM.pdf]

Supplementary Information

	Page
**S1.** ICD-10 codes used to identify deaths from colorectal cancer	2
**S2.** Flow diagram of study population	3
**S3.** Descriptive statistics for predictor variables by linkage status	4
**S4.** Methods for imputed datasets	5
**S5.** Sensitivity analyses	6
**S6.** Comparison of patients by missing data status	7
**S7.** How to calculate probabilities of colorectal cancer death	8

Supplement S1. ICD-10 codes used to identify deaths from colorectal cancer

**ICD-10 code**	**Description**
C18	Malignant neoplasm of colon
C19	Malignant neoplasm of rectosigmoid junction
C20	Malignant neoplasm of rectum
C26.0	Malignant neoplasm of other and ill-defined digestive organs: intestinal tract, part unspecified
C26.9	Malignant neoplasm of other and ill-defined digestive organs: ill-defined sites within the digestive system
C76.2	Malignant neoplasm of other and ill-defined sites: abdomen
C77	Secondary and unspecified malignant neoplasm of lymph nodes
C78	Secondary malignant neoplasm of respiratory and digestive organs
C79	Secondary malignant neoplasm of other and unspecified sites
D37.4	Neoplasm of uncertain or unknown behaviour of oral cavity and digestive organs: colon
D37.5	Neoplasm of uncertain or unknown behaviour of oral cavity and digestive organs: rectum
D37.7	Neoplasm of uncertain or unknown behaviour of oral cavity and digestive organs: other digestive organs

Supplement S2. Flow diagram of study population

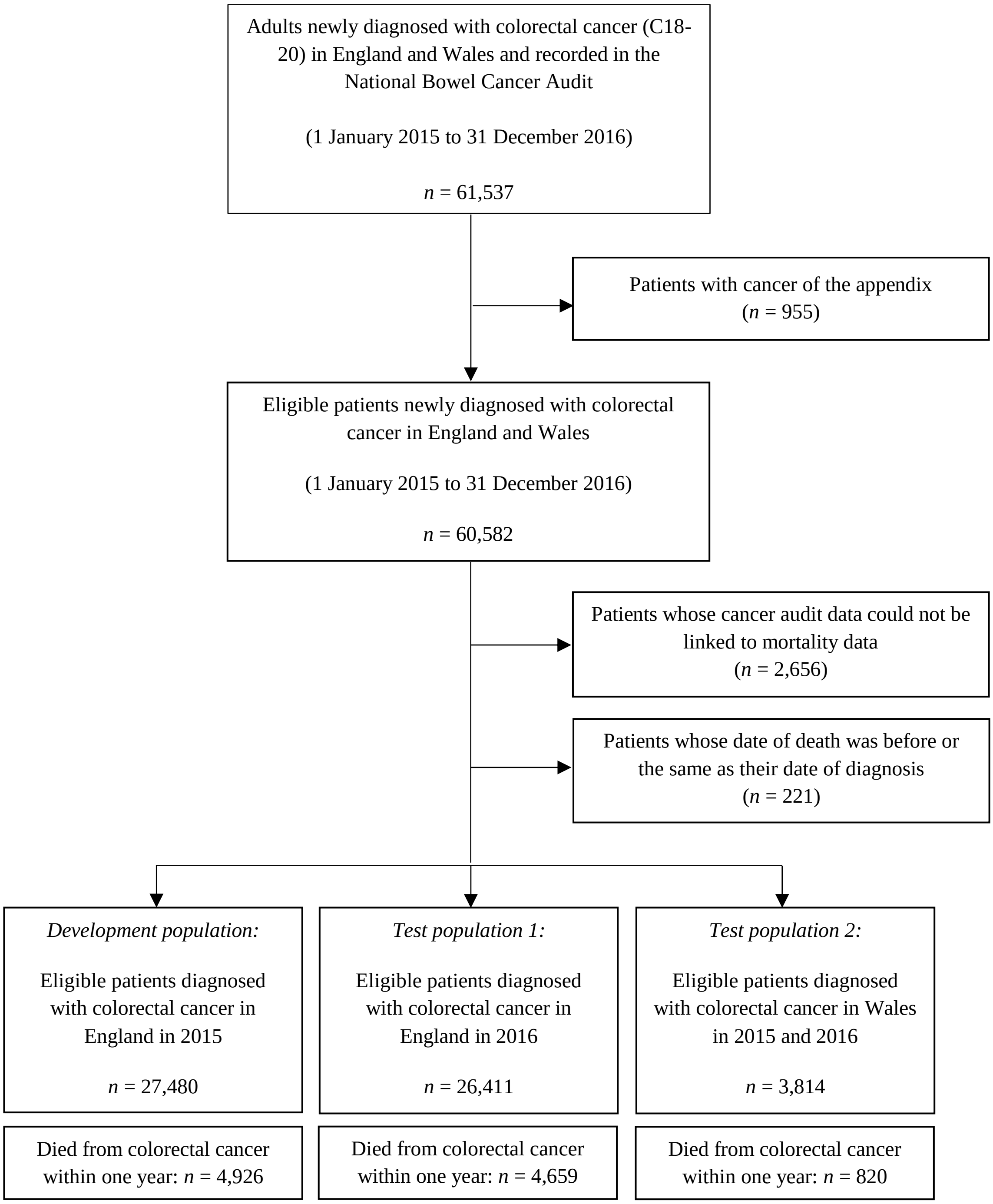

Supplement S3. Descriptive statistics for predictor variables by linkage of the National Bowel Cancer Audit and Office for National Statistics death records

	**Linked patients (*n*=57 926)**	**Unlinked patients (*n*=2656)**
**Median age (years; IQR)***	72 (63 to 80)	73 (65 to 80)
**Gender (%)**		
Male (*vs.* female)	56.9	54.3
**Median socioeconomic status (IQR)†**	0.1 (-1.0 to 1.1)	0.1 (-0.8 to 1.0)
**Referral source (%)**		
Emergency admission	13.9	14.4
Urgent care/ED visit	2.8	2.6
Screening	10.2	9.1
Primary care	55.5	55.8
Other	17.6	18.1
**Performance status‡ (%)**		
0 (fully active)	44.6	43.0
1	30.9	31.6
2	15.2	15.7
3	7.9	7.8
4 (completely disabled)	1.5	1.9
**Tumour site (%)**		
Caecum	14.8	16.6
Ascending colon	11.0	10.8
Hepatic flexure	4.0	4.2
Transverse colon	6.5	6.9
Splenic flexure	2.6	3.3
Descending colon	3.5	4.0
Sigmoid colon	23.2	22.5
Rectosigmoid junction	5.6	5.2
Rectum	28.7	26.5
**T-stage (%)**		
1	5.9	6.1
2	19.9	19.9
3	51.7	51.6
4	22.4	22.5
**N-stage (%)**		
0	47.8	47.9
1	34.1	35.1
2/3	18.1	17.0
**M-stage (%)**		
0	77.9	78.4
1	22.1	21.6
**Treatment intent (%)**		
Curative	73.4	75.7
Non-curative	20.6	18.1
No active cancer treatment	6.0	6.2

*Age range was 18 to 104. †Rescaled national rank of the area in which a patient resided (0 is the median; -1 is the lower quartile; 1 is the upper quartile). ‡Measured on the Eastern Cooperative Oncology Group (ECOG) scale. ED: emergency department.

Supplement S4. Methods for imputed datasets

We applied multiple imputation using chained equations to predict missing values of predictor variables.[1] For each of these variables, all other predictors, colorectal cancer death status and the Nelson-Aalen estimate of the cumulative baseline hazard were included in the imputation model.[2] The imputation method was predictive mean matching for continuous variables, logistic regression for binary variables, and polytomous regression for categorical variables with more than two categories. We generated 40 complete imputed datasets for each population, following guidance to use at least as many imputed datasets as the percentage of observations with incomplete data.[3] Imputation was done separately for each population to avoid introducing artificial correlations across populations.

Estimates from Cox proportional hazards regression in each imputed dataset were pooled using Rubin's rules.[4] When assessing the internal validity of the model using 10-fold cross-validation, we calculated the medians of the cross-validated estimates of the Brier score and *c*-index in each imputed dataset.[5] As there were 40 complete imputed datasets for the development population and each test population, there were 1,600 (40 x 40) unique combinations of datasets for each test population. When assessing external validity, we tested model performance for each of these combinations, then calculated the medians of the Brier score and *c*-index across the 1,600 combinations.

For the calibration plots, we calculated the mean of the complementary log-log transformation of the probability estimated for each patient across the 1,600 combinations.[5] The back-transformed means were then plotted against the observed risks.

References:

1. van Buuren S, Groothuis-Oudshoorn K. mice: Multivariate Imputation by Chained Equations in R. *Journal of Statistical Software* 2011;45(3):67.

2. White IR, Royston P. Imputing missing covariate values for the Cox model. *Stat Med* 2009;28(15):1982-98.

3. White IR, Royston P, Wood AM. Multiple imputation using chained equations: Issues and guidance for practice. *Stat Med* 2011;30(4):377-99.

4. Rubin DB. *Multiple Imputation for Nonresponse in Surveys*. New York: Wiley; 1987.

5. Marshall A, Altman DG, Holder RL, Royston P. Combining estimates of interest in prognostic modelling studies after multiple imputation: current practice and guidelines. *BMC Med Res Methodol* 2009;9:57.

Supplement S5. Sensitivity analyses

We examined whether adding an interaction term between patient age and M-stage improved model performance, as this interaction was predictive of 90-day postoperative mortality in a similar dataset.[1] We also added interactions between age and treatment intent and between M-stage and treatment intent in this model, to allow treatment to moderate key patient characteristics.

We assessed the benefit of adding two extra variables as predictors: a comorbidity score and the number of unplanned hospital admissions in the year before diagnosis. These variables were limited to sensitivity analyses as they are not available from the same dataset as the other predictors so would be more difficult to implement practically. The comorbidity score was a weighted number of comorbidities, based on 28 non-cancer comorbidities[2] and their established weights.[3] To generate the scores, we linked National Bowel Cancer Audit records to routine hospital records of inpatient admissions (Hospital Episode Statistics[4]). We used ICD-10 codes[5] to identify each comorbidity in the year before diagnosis. The number of unplanned admissions was derived from the same dataset.

The Cox proportional hazards model assumes that the hazard ratios are constant over the follow-up period. We explored whether different follow-up periods affected the hazard ratios estimated by censoring survival times beyond 365 days at 365 days and re-estimating the model.

References:

1. Walker K, Finan PJ, van der Meulen JH. Model for risk adjustment of postoperative mortality in patients with colorectal cancer. *Br J Surg* 2015;102(3):269-80.

2. Elixhauser A, Steiner C, Harris DR, Coffey RN. Comorbidity measures for use with administrative data. *Med Care* 1998;36(1):8-27.

3. van Walraven C, Austin PC, Jennings A, Quan H, Forster AJ. A Modification of the Elixhauser Comorbidity Measures into a Point System for Hospital Death Using Administrative Data. *Med Care* 2009;47(6):626-33.

4. NHS Digital. *Hospital Episode Statistics (HES)*. Available from: https://digital.nhs.uk/data-and-information/data-tools-and-services/data-services/hospital-episode-statistics.

5. Quan H, Sundararajan V, Halfon P, Fong A, Burnand B, Luthi JC, et al. Coding algorithms for defining comorbidities in ICD-9-CM and ICD-10 administrative data. *Med Care* 2005;43(11):1130-9.

Supplement S6. Comparison of patients with completely observed data for all variables and patients with missing data for one or more variables

	Completely observed data	Missing data
**Number (%) of patients**	35 472 (61.5)	22 233 (38.5)
**Median survival/follow-up time (IQR)**	597 (406 to 823)	563 (318 to 816)
**Status at follow-up end, *n* (%)**		
Survived	25 053 (70.6)	13 679 (61.5)
Colorectal cancer death	8524 (24.0)	6861 (30.9)
Other death	1895 (5.3)	1693 (7.6)
**Median age (years; IQR)***	72 (63 to 80)	73 (64 to 81)
**Gender, *n* (%)**		
Male (*vs.* female)	20 480 (57.7)	12 335 (55.5)
**Median socioeconomic status (IQR)†**	0.1 (-1.0 to 1.1)	0.0 (-1.0 to 1.0)
**Referral source, *n* (%)**		
Emergency admission	4297 (12.1)	3561 (16.6)
Urgent care/ED visit	772 (2.2)	792 (3.7)
Screening	3704 (10.4)	2144 (10.0)
Primary care	20 706 (58.4)	10 941 (51.1)
Other	5993 (16.9)	3993 (18.6)
**Performance status, *n* (%)‡**		
0 (fully active)	16 366 (46.1)	5099 (40.7)
1	10 985 (31.0)	3875 (30.9)
2	5309 (15.0)	1943 (15.5)
3	2425 (6.8)	1331 (10.6)
4 (completely disabled)	387 (1.1)	295 (2.4)
**Tumour site, *n* (%)**		
Caecum	5069 (14.3)	3477 (15.6)
Ascending colon	3974 (11.2)	2393 (10.8)
Hepatic flexure	1386 (3.9)	923 (4.2)
Transverse colon	2198 (6.2)	1542 (6.9)
Splenic flexure	857 (2.4)	606 (2.7)
Descending colon	1225 (3.5)	819 (3.7)
Sigmoid colon	7639 (21.5)	5756 (25.9)
Rectosigmoid junction	2057 (5.8)	1191 (5.4)
Rectum	11 067 (31.2)	5526 (24.9)
**T-stage, *n* (%)**		
1	2088 (5.9)	683 (6.1)
2	7174 (20.2)	2118 (19.0)
3	18 476 (52.1)	5674 (50.9)
4	7734 (21.8)	2684 (24.1)
**N-stage, *n* (%)**		
0	16 276 (45.9)	6600 (53.1)
1	12 621 (35.6)	3710 (29.9)
2/3	6575 (18.5)	2109 (17.0)
**M-stage, *n* (%)**		
0	28 312 (79.8)	9929 (73.2)
1	7160 (20.2)	3627 (26.8)
**Treatment intent, *n* (%)**		
Curative	27 176 (76.6)	12 397 (67.5)
Non-curative	6740 (19.0)	4291 (23.4)
No active cancer treatment	1556 (4.4)	1671 (9.1)

*Age range was 18 to 104. †Rescaled national rank of the area in which a patient resided (0 is the median; -1 is the lower quartile, more deprived; 1 is the upper quartile, less deprived). ‡Measured on the Eastern Cooperative Oncology Group (ECOG) scale. ED: emergency department; IQR: interquartile range.

Supplement S7. How to calculate probabilities of colorectal cancer death within 90, 180, and 365 days of diagnosis from the prediction model

The probability of colorectal cancer death by time *t* equals: $1 - \ S_0(t)^{\exp(\beta_1 X_1 + \ \beta_2 X_2 + \ \cdots \ + \ \beta_p X_p)}$.

where $S_0(t)$ is the baseline survival probability at time t, β are the regression coefficients from the estimated Cox proportional hazards regression model, X are predictor variables, and p is the number of predictor variables.

The value of $S_0(t)$ for an individual with all risk factors equal to zero** is:

- For the 90 days period: 0.9983142
- For the 180 days period: 0.9969342
- For the 365 days period: 0.9941971

The values of β for each predictor variable are:

	**β**
**Age (per 10 years)***	0.18802794
**Gender**	
Male	0
Female	0.02453517
**Socioeconomic status†**	-0.03625044
**Referral source**	
Emergency admission	0
Urgent care/ED visit	-0.01889659
Screening	-1.09412094
Primary care	-0.31258302
Other	-0.28886696
**Performance status‡**	
0 (fully active)	0
1	0.18617150
2	0.42744336
3	0.84882276
4 (completely disabled)	1.21503157
**Tumour site**	
Caecum	0
Ascending colon	-0.08209810
Hepatic flexure	0.13092154
Transverse colon	0.07391412
Splenic flexure	-0.12596959
Descending colon	-0.08558521
Sigmoid colon	-0.21539719
Rectosigmoid junction	-0.16826741
Rectum	-0.23575824
**T-stage**	
1	0
2	0.70853229
3	1.10545928
4	1.54166050
**N-stage**	
0	0
1	0.14132215
2/3	0.42053813
**M-stage**	
0	0
1	1.03367028
**Treatment intent**	
Curative	0
Non-curative	1.34685236
No active cancer treatment	1.34679083

*Age range was 18 to 104. †Rescaled national rank of the area in which a patient resided (0 is the median; -1 is the lower quartile, more deprived; 1 is the upper quartile, less deprived). ‡Measured on the Eastern Cooperative Oncology Group (ECOG) scale. ED: emergency department. **Baseline survival probabilities assume age zero and median socioeconomic status.
